# A systematic review of prevalence and predictors of depression in systemic sclerosis based on the CES-D, BDI, and PHQ- 9 self-assessment questionnaires

**DOI:** 10.1007/s10067-025-07440-w

**Published:** 2025-04-16

**Authors:** Esra Mehmetoglu, Anvitha Mummadisetty, Andreas Chatzittofis, Konstantinos Parperis, Nora Sandorfi, Chris T. Derk

**Affiliations:** 1 Istanbul Faculty of Medicine, Istanbul, Turkey; 2 Kakatiya Medical College, Warangal, India; 3 Department of Psychiatry, University of Cyprus Medical School, Nicosia, Cyprus; 4 Department of Internal Medicine, Division of Rheumatology, University of Cyprus Medical School, Nicosia, Cyprus; 5 Department of Internal Medicine, Division of Rheumatology, University of Philadelphia, Philadelphia, PA 19014 USA; 6 Division of Rheumatology, University of Pennsylvania, 5 th Floor White Bldg, 3400 Spruce Street, Philadelphia, PA 19014 USA

**Keywords:** Depressive disorder, Depression, Scleroderma, Systemic sclerosis

## Abstract

**Objectives:**

This study aimed to investigate the prevalence of depression in Systemic Sclerosis (SSc) patients using depression self-assessment tools such as the Beck Depression Inventory (BDI), the Centre for Epidemiologic Studies Depression Scale (CES-D), and the Patient Health Questionnaire (PHQ-9) and to explore the clinical characteristics of SSc patients with depression and identify potential risk factors for depression.

**Methods:**

Based on PRISMA guidelines, an electronic search was conducted in PubMed, Embase, PsycINFO, and Google Scholar to collect studies assessing systemic sclerosis and depression up to April 2024. Original research studies in SSc patients and depression using BDI > 10, CES-D > 16, and PHQ-9 > 10 that met our inclusion criteria were included and appraised using the Joanna Briggs Institute (JBI) instrument, then data extraction was performed.

**Results:**

We identified 497 articles, of which 22 were included in this systematic review. Among the 4,165 patients who completed the self-assessment questionnaires, 1486 (35.6%) met the criteria for depression where 564 (31.9%) of patients were identified based on the CES-D, 410 (55.1%) from the BDI, and 512 (30.8%) from the PHQ-9 group. Gastrointestinal involvement was the most identified predictor for depression, while pain, disease activity, and pulmonary and joint symptoms of SSc were also commonly associated with depression. Non-SSc-related predictors of depression included unemployment, low educational level, and unmarried status.

**Conclusion:**

Higher prevalence of depression is seen among patients with SSc. Although the prevalence varies according to the assessment tool used, we found correlation of depression estimates in SSc-patients between the CES-D and PHQ-9 scores. Moreover, this review identifies the significant predictors of depression in SSc patients and highlights the need of mental healthcare professionals to engage in the care of SSc patients.

**Conclusion:**

HB level was significantly related to disease activity and structural damage in RA patients.

**Table:** 

**Key Points** • *There is significant variability in estimates of depression among different self-assessment questionnaires in patients with SSc.* • *We found correlation of depression estimates in SSc-patients between the CES-D and PHQ9 scores.* • *Among the studies reviewed strong predictors of depression in SSc-patients were identified. These were significant GI and arthritic manifestations, increased pain and disease severity, and a lower educational level*. • *Importance in engaging mental healthcare professionals in the care of SSc- patients as per our study up to a third of patients may benefit from this*.

## Introduction

Systemic sclerosis (SSc) is a rare autoimmune rheumatic disease characterized by vascular dysfunction, autoimmunity, and progressive fibrosis affecting the skin and internal organs. The disease is classified into two subtypes: limited cutaneous SSc (lcSSc) and diffuse cutaneous SSc (dcSSc) [1, 2].

High rates of depression are commonly observed among patients with chronic illness, and this may in turn impact physical health, cognitive function, and social processes [3]. Individuals with chronic connective tissue diseases, including SSc, frequently face mental health challenges [4]. Depression, a complex and difficult-to-manage condition, is one of the most common mood disorders experienced by SSc patients. This mood disorder is presumed to be a consequence of the disease’s progressive, disfiguring, and debilitating course [5, 6] with variable prevalence across studies. This discrepancy may be a result of the heterogeneity of the self-assessment tools being used to screen for depressive symptoms as compared to a standardized, comprehensive exam by a mental health professional.

The first study to inquire about the prevalence of depression in SSc was conducted by Roca et al. [7] in 1996 where they used the BDI [8] self-assessment questionnaire and found nearly two-thirds of SSc patients had depressive symptoms. In a more recent systematic review, Thombs et al. [9] examined studies that used different self-assessment depression questionnaires and reported that the prevalence ranged from 36 to 65% in SSc patients. Further research has shown that depression is more prevalent in patients with SSc compared to those with other rheumatic diseases [10, 11]. This highlights the significant impact of SSc on patients' mood, underscoring the need for careful consideration of mental health in daily clinical practice. Based on existing data, physical changes [12], impaired physical function [13, 14] and neuronal-immunologic alterations [15, 16] caused by SSc have been described as predictors of depression in SSc patients. There are conflicting findings regarding the association between disease severity and depressive symptoms in SSc. One study reported a significant relationship, while other studies did not find such an association [5, 17, 18].

Patients suffer from various cutaneous problems such as skin thickening, skin pigmentation changes, Raynaud’s, digital ulcers, poikiloderma, telangiectasias, and more specifically changes in their facial characteristics and the appearance of their hands. These alterations can be very distressing for patients [19, 20], and they mention skin deformity as one of the main stressors of their disease [21]. They further report that they worry about changes in their appearance, especially in socially significant parts of the body [20]. One study indicated that disfigurements led to body image dissatisfaction in women with SSc which was associated with depressive symptoms [22].

A high mortality and morbidity rate and an unpredictable course of the disease are also significant determinants of how patients feel. When unmet needs were considered, patients frequently expressed concern about disease worsening and uncertainty about the future [23]. In another study, worries about future outcomes were a noteworthy burden [20] and research by Kwakkenbos et al. (2012) [24] showed that increased fear of disease progression was associated with more depressive symptoms. The illness-related uncertainty in terms of variability in disease progression was also found to be a predictor of poor psychosocial adjustment to the illness [25] which in turn correlated with symptoms of depression [7].

A wide range of self-assessment screening tools have been used in different research studies aimed at estimating the prevalence and predictors of depression in SSc patients. Although many screening tools are available, this study focuses on three commonly used screening instruments: the BDI, the CES-D) [26], and the PHQ-9 [27]. These self-assessment measures are widely used to assess depressive symptoms and depression severity and have been extensively applied in rheumatologic disorders [28]. Each has demonstrated strong utility in screening for depression and exhibits good predictive validity among patients with SSc [7, 29, 30].

These questionnaires have both cognitive-affective and somatic symptoms; thus, it is important to differentiate potential depression from physical symptoms related to SSc. In a few studies, somatic symptoms were assessed separately to prevent the bias caused by the overlap of depressive symptoms with symptoms of SSc. Results have shown that cognitive-affective depressive symptoms and total BDI correlated well [7, 31]. Nietert et al. [14] also found relatively unaffected results when excluding somatic symptoms from the CES-D which was consistent with the results of the study by Thombs et al. [32] that showed that the degree of overlapping bias is negligible.

The objective of our study was to use a systematic review methodology to investigate the prevalence of depression in SSc patients using depression self-assessment tools. These tools include the BDI with a cutoff score above 10, the CES-D with a cutoff score above 16, and the PHQ-9 with a cutoff score above 10. The study also aimed to explore the clinical characteristics of SSc patients with depression and identify risk factors that are associated with depression in SSc patients.

## Patients and methods

### Search strategy

**Fig. 1:**
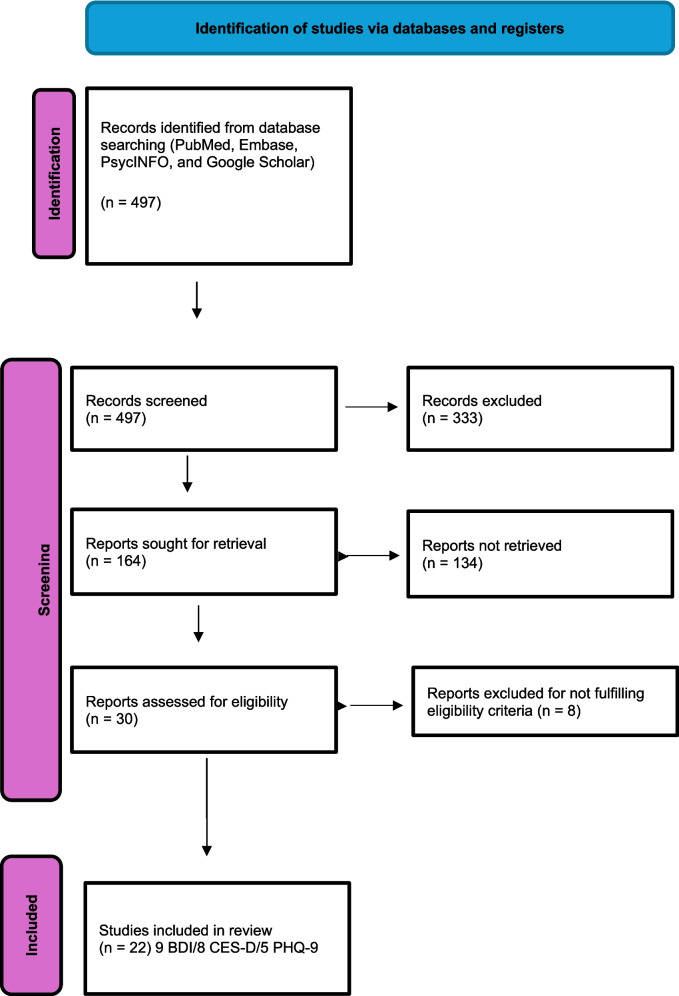
PRISMA flow diagram showing the inclusion and exclusion process

This is a systematic review following the PRISMA guidelines [33]. The search plan included doing an electronic search of PubMed, Embase, PsycINFO, and Google Scholar with the words Systemic Sclerosis OR Scleroderma OR Scleroderma, Systemic AND depressive disorder OR depression OR depressive symptoms up to April of 2024. We did an initial screen by title and abstract to exclude papers not relevant to our study, and we then proceeded in the next cycle with a full-text screen to exclude studies that did not meet our inclusion and exclusion criteria. The references of the papers included in this systematic review were also reviewed to look for additional original work that may not have been found in our original search. The next step involved reviewing all the selected articles under all three assessment tools and coming to a final consensus as to which studies to include (Fig. 1).

### Study selection

We included published studies of original research in any language that used the BDI, CES-D, and PHQ-9 to determine the prevalence of depression in SSc patients.

The BDI has 21 self-administered questions on a 4-point Likert-type scale ranging from 0 to 3 based on the severity of symptoms over the past 2 weeks. The total score can range from 0 to 63 with higher scores indicating more severe depressive symptoms. It is composed of items that relate to symptoms of depression such as hopelessness, irritability, and guilt, as well as physical symptoms such as fatigue, weight loss, and lack of interest in sex [8]. The CES-D has 20 self-administered questions on a 4-point Likert-type scale ranging from 0 to 3 based on occurrences over the past week. The total score can range from 0 to 60, the higher the score the greater the depressive symptoms. It is composed of items that mostly relate to the affective component of depression [26]. The PHQ-9 has 9 self-administered questions also on a Likert-type scale ranging from 0 to 3 over the past 2 weeks. The total score can range from 0 to 27, the higher the score the greater the depressive symptoms. It is composed of both affective and physical symptoms that relate to depression such as hopelessness, lack of interest, poor appetite, and trouble falling asleep [27].

For this systematic review, we picked cut-offs for the different self-reported tools based on what was previously used by most of the studies. A cut-off of 10 is used to assess mild depressive mood in the BDI, 16 to assess for probable depression in the CES-D, and 10 to assess for moderate depression in the PHQ-9.

Inclusion criteria were original research studies in SSc patients and depression using BDI > 10, the CES-D > 16, and the PHQ-9 > 10. Exclusion criteria were abstracts, case reports, case series, systematic or narrative reviews, meta-analyses, studies using different self-assessment tools or cut-off points, duplicate studies, and studies that did not evaluate the prevalence of depression in SSc patients. The initial database search was conducted by 2 of the authors (E.M., A.M.). In the next step, an abstract and title screen on the results were done to exclude further studies (E.M., A.M., C.T.D.). All 6 authors performed a full-text review based on inclusion and exclusion criteria. Four authors (C.T.D, N.S., A.C, K.P.) split into 2 teams to hand-search articles, focusing on BDI, CES-D, and PHQ-9 studies. The teams conducted another full-text review and went through the inclusion and exclusion criteria. Discrepancies between reviewers in each team regarding article selection were resolved by consensus. Once the two teams selected the articles to be included, all four reviewers from the last cycle reviewed the selected articles and came to a final consensus [5, 7, 13, 14, 17, 18, 22, 24, 31, 34–46] (Fig. 1). We did not use automation tools in the process.

### Data extraction and assessment of methodologic quality of study

**Fig. 2:**
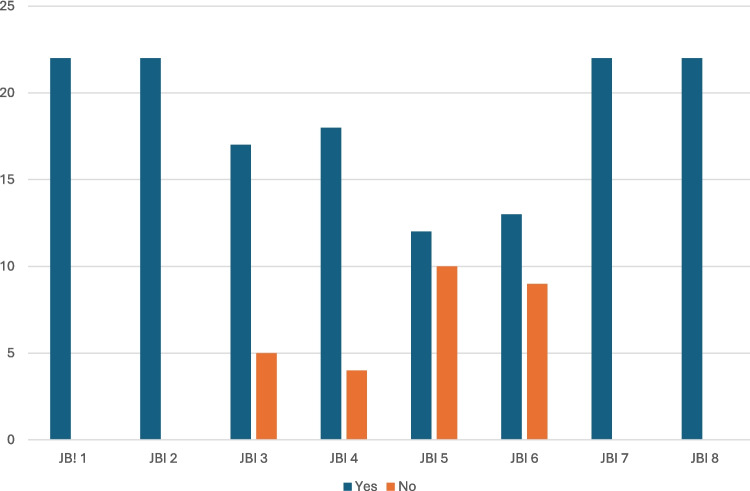
JBI Critical appraisal checklist of systemic review articles

From the selected studies, we extracted data on the year of publication, the self-administered instrument used and its cut-off value, the country where the work was done, and the number of SSc patients who took the self-administered questionnaire. We documented how each study classified SSc patients, using either the 1980 American Rheumatism Association (ARA) criteria [47], the 2013 American College of Rheumatology/European League Against Rheumatism (ACR/EULAR) criteria [48], or other classification methods. For demographics, we collected data on the mean age of the study population and, where available, the mean age of patients diagnosed with depression. We gathered data on gender, SSc subtype, the disease duration, and the mean modified Rodnan skin (mRss) score. The number and percentage of patients diagnosed with depression were noted, along with any SSc-related or non-SSc-related predictors of depression, if reported. The Joanna Briggs Institute (JBI) instrument [49] was used to evaluate the methodological quality of the selected studies. This tool consists of eight key items: defined inclusion criteria, detailed study subjects and setting, valid exposure measurement, objective condition measurement, confounding factors identification and management, valid outcome measurement, and appropriate statistical analysis. Each item can get a score of 0 (no, unclear, or not applicable) or 1 (yes). In Table 1, we described the total score for each article used in this review and this score can range from 0 to 8 and in Fig. 2 we described the total score for each of the 8 items of the checklist for all the studies used in this review and a total score of 22 is distributed among the 0 and 1 scores for each item.

**Table 1:** Articles used for the systematic review evaluating prevalence and predictors of depression in SSc patients

Study	Type of Study	Assessment tool for depressive symptoms: cutoff	Country	Classification criteria for SSc	SSc patients with depression (n)	All SSc patients (n)	SSc patients with depression (%)	Age of SSc with depression (mean and SD)	Age of the total study population (mean and SD)	Female (%)	dcSSc (%)	Disease duration years (mean and SD)	mRSS in patients with SSc and depression (mean and SD)	mRSS in total study population (mean and SD)	Predictors of depression SSc related Significant multivariate (*p* < 0.05)	Predictors of depression non-SSc related Significant multivariate (*p* < 0.05)	JBI score
Kwakkenbos [24]	Cohort study	CES-D > = 1 6	Netherland s	ARA	69	215	32.1%	N/A	56.4 + 12.0	67.9%	26.5%	9.2 ± 7.9	N/A	6.4 + 5.9	Pain, fatigue, fear of progression	Satisfaction with social support, emotion-focused coping, helplessness	8
Poole [34]	Cross-sectional study	CES-D > = 1 6	USA	N/A	56	83	68%	N/A	53.4 + 10.8	94%	46.9%	9.5 ± 7.2	N/A	N/A	N/A	Lower service to others, less domestic chores	5
Savoie [35]	Cohort study	CES-D > = 1 6	USA	ACR/EUL AR	38	129	29%	59	62	89%	38%	9	6	4	Higher baseline dyspnea index, mRSS score, UCLA-ST C GIT score	N/A	8
Heyne [36]	Cross-sectional study	CES-D > = 1 6	Germany	ACR/EUL AR	33	79	42.3%	N/A	61.5 + 12.6	81%	16.5%	12.1 ± 10.3	N/A	5.1 + 6.1	PGA, pain, pulmonary HTN, muscle weakness, dyspnea, treated with immunosuppression, HAQ score	N/A	8
Thombs [17]	Cross-sectional study	CES-D > = 1 6	Canada	N/A	132	376	35.1%	53.4 ± 12.6	55.4 ± 12.7	87.2%	48.4%	10.8 ± 8.7	12.1 ± 11.0	11.2 ± 10.4	Higher physician-rate d overall disease severity, more tender joints, more GI symptoms, more difficulty breathing	Less education, not married	7
Nietert [14]	Cross-sectional study	CES-D > = 1 6	USA	ARA	26	72	36.1%	N/A	51.3 + 12.4	80.6%	61.1	6.2 ± 7.6	N/A	16.6 + 12.2	More disability, worse GI functional status (both upper and lower GI)	Less education	8
Golemati [37]	Case–control study	CES-D > = 1 6	Greece	ARA	41	85	48.2%	N/A	53.3 + 13.6	96.4%	67.1%	9.2 + 7.6	N/A	N/A	Disease activity, total skin score, PAH, FVC score	Lower education, lower extraversion, neuroticism, lower seeking of social support, negative reappraisal, less social support satisfaction , more negative life events	8
Levis [38]	Cross-sectional study	CES-D > = 1 6	Canada	ACR/EUL AR	269	725	37%	N/A	57.4 + 11.2	100%	40.7%	17.6 ± 12.3	N/A	7.9 + 8.5	Tender joints, gastrointestinal involvement	Unmarried, low marital satisfaction, less than a high school education	7
Tedeschini [31]	Cross-sectional study	BDI > = 10	Italy	ARA	36	78	46.2%	55.7 ± 11.6	53.7 ± 12.1	92.3%	16.7%	7.7 ± 6.4	9.3 ± 6.6	8.6 ± 6.7	Increased disability, pain, disease activity, articular involvement	Unemployment	8
Matsuura [18]	Cross-sectional study	BDI > 10	Japan	ARA	23	50	46%	N/A	59.6 + 11.2	82%	40%	13.6	N/A	11.8 + 7.3	N/A	Low sense of coherence, helplessness	8
Benrud-Larson [13]	Cross-sectional study	BDI > = 10	USA	N/A	72	142	51%	N/A	52.1 + 13.7	91%	35%	N/A	N/A	N/A	N/A	N/A	7
Ostojic [5]	Case–control study	BDI > = 10	Serbia	ARA	24	35	68.6%	N/A	N/A	77.1%	34.3%	7.5	N/A	N/A	Longer disease duration, pain	Female, older, unemployed, retired	6
Roca [7]	Cross-sectional study	BDI > = 10	USA	ARA	35	54	64.8%	N/A	N/A	85%	46.3%	N/A	N/A	N/A	Younger patients, presence of digital ulcers, greater self-related functional impairment (HAQ score)	N/A	7
Benrud-Larson [22]	Cross-sectional study	BDI > = 10	USA	N/A	64	127	50%	N/A	52.8 + 13.7	100%	33.1%	N/A	N/A	N/A	N/A	N/A	6
Bagnato [39]	Cross-sectional study	BDI > = 10	Italy	ACR/EUL AR	16	33	48.4%	N/A	56.1 + 11.5	100%	38%	7.1 + 6.7		17.9 + 9	N/A	N/A	5
Beretta [40]	Cross-sectional study	BDI > = 10	Italy	ARA	62	111	55.8%	N/A	56.4 + 11.4	N/A	24.3%	10.9 + 7.3	N/A	6.1 + 5.3	N/A	N/A	8
Faezi [41]	Cross-sectional study	BDI > = 10	Iran	ARA	78	114	68.4%	N/A	N/A	88.6%	55.3%	N/A	N/A	17.64 + 9.07	dcSSc, higher mRSS, pulmonary and gastrointestinal manifestations	N/A	5
Kong [42]	Cross-sectional study	PHQ9 > 10	China	ACR/EUL AR	106	273	43.59%	N/A	45	90.5%	N/A	6	N/A	N/A	N/A	N/A	7
Carandina [43]	Cross-sectional study	PHQ9 > 10	Italy	ACR/EUL AR	9	20	45%	N/A	56	80%	20%	13	N/A	N/A	N/A	N/A	6
Wafki [44]	Cross-sectional study	PHQ9 > 10	Morocco	ARA	46	59	77.4%	N/A	49.5 + 13	50.7%	96%	9 + 4.5	N/A	N/A	Prolonged disease, severe joint pain, disease severity, elevated inflammatory markers	N/A	5
Razykov [45]	Cross-sectional study	PHQ9 > 10	Canada	N/A	149	345	43.1%	N/A	57.7 + 11.8	87.5%	24.3%	9.7 + 8.2	N/A	8.3 + 9.6	N/A	N/A	5
Arthurs [46]	Cross-sectional study	PHQ9 > 10	Canada	N/A	202	960	21.4%	N/A	56.6 + 11.5	87%	31.4%	N/A	N/A	9.7 + 9.0	N/A	N/A	6

*SSc*  systemic sclerosis, *SD*  standard deviation, *dcSSc*  diffuse cutaneous systemic sclerosis, *JBI*  Joanna Briggs Institute, *ARA*  American Rheumatism Association, *N/A*  not available, *ACR*  American College of Rheumatology, *EULAR*  European League Against Rheumatism, *UCLA SCTC GI*  University of California in Los Angeles Clinical Trails Consortium Gastrointestinal score, *mRSS*  modified Rodnan skin score, *PGA*  physical global assessment, *HTN*  hypertension, *HAQ*  Health Assessment Questionnaire, *BDI*  Beck Depression Inventory, *PHQ-9*  nine-item Patient Health Questionnaire, *CES-D*  Center for Epidemiological Studies- Depression scale, *PGA* patient global assessment, *GI* gastrointestinal, *FVC* forced vital capacity, *PAH* pulmonary arterial hypertension

## Results

### Demographics

In this systematic review, we included 22 published studies that met our inclusion and exclusion criteria, encompassing a total of 4,165 patients who completed the self-assessment questionnaires (1764 for the CES-D, 744 for the BDI, and 1657 for the PHQ-9). Among the SSc patients who participated in the self-assessment questionnaires 3,658 (90.2%) were female. Notably one study did not provide gender information of its population. The mean age was 52.0 years, and the mean disease duration was 9.8 years with 5 studies not defining this. The mean skin score based on the mRss was 8.7 and 1,469 (37.7%) patients had dcSSc, with one study not defining this. The studies included in the systematic review included eighteen cross sectional studies, two case control studies and two cohort studies. Six of the studies took place in the United States, four in Canada, and four in Italy, while each one of the other studies came from a different country (Table 1).

### Prevalence of depression

Based on the self-assessment questionnaires, 1486 (35.6%) of SSc patients across all studies met the criteria for depression. This includes 564 (31.9%) of these patients from the CES-D group, 410 (55.1%) from the BDI group and 512 (30.8%) from the PHQ-9 group.

### Predictors of depression

Our systematic review identified that gastrointestinal symptoms were the most associated, SSc-related symptom, with depression, while pain, disease activity and joint symptoms of SSc were the second most associated with depression, all of them reaching statistical significance in multiple studies. Pulmonary symptoms were studied in many ways across the different studies as predictors of depression, based on subjective symptoms and objective measurements such as the FVC, when they were all lumped in the same category, they were the most commonly involved with depression but since the descriptions and measurements were different from study to study, we could not clearly conclude this. The most associated non-SSc related predictor of depression was low educational level. All the different predictors of depression which reached statistical significance in the articles included in this systematic review are presented in Table 2.

**Table 2:** Predictors of depression in SSc which reached statistical significance in the articles included in the systematic review

	Predictors	Total number of studies
SSc related	Pain	5
	Disease severity/Disability	4
	Fatigue	1
	Fear of progression	1
	Pulmonary involvement (Higher baseline dyspnea index, dyspnea, Pulmonary HTN, PAH, FVC score, difficulty breathing)	6
	mRSS score	3
	GIS involvement (UCLA-ST GIT score, worse status)	5
	Longer disease duration	2
	Digital ulcers	1
	dcSSc	1
	Elevated inflammatory markers	1
	HAQ score	2
	Joint involvement	4
	Muscle weakness	1
	Treated with immunosuppression	1
	Patient global assesment	1
Non-SSc related	Lower service to others	1
	Less domestic chores	1
	Lower extraversion	1
	Neuroticism	1
	Negative reappraisal	1
	More negative life events	1
	Social support satisfaction	2
	Emotion-focused coping	1
	Helplessness	2
	Less education	4
	Unmarried	2
	Unemployment/retired	2
	Marital satisfaction	1
	Older	1
	Younger	1

*SSc*  systemic sclerosis, *FVC* forced vital capacity, *GIS* gastrointestinal system, *UCLA SCTC GI*  University of California in Los Angeles Clinical Trails Consortium Gastrointestinal score, *mRSS*  modified Rodnan skin score, *dcSSc*  diffuse cutaneous systemic sclerosis, *HAQ* Health Assessment Questionnaire, *HTN* hypertension, *PAH* pulmonary arterial hypertension

In a study by Kwakkenbos L et al. [24] pain and fatigue were independently associated with depressive characteristics, and when adding psychological factors, satisfaction with social support, higher fear of progression, and emotion-focused coping and helplessness were also significantly associated with depressive symptoms. Furthermore, Poole JL et al. [34] looked at depression in SSc patients and how it relates to participation in life situations. Domestic chores, household maintenance, service to others, and societal activities were the 4 domains looked at. They found that lower service to others and less domestic chores were related to higher depressive symptoms. In another study by Savoie MB et al. [35] a higher baseline of dyspnea index, a higher mRSS, and a higher University of California Los Angeles Scleroderma Clinical Trial Consortium Gastrointestinal score (UCLA-SCTC GIT score) were associated with higher CES-D scores, i.e., depression.

A study from a Dermatology Department in Dresden Germany [36] showed that worse physician global (PGA) scores, pulmonary hypertension, muscle weakness, worse HAQ score, and treatment with immunosuppressive drugs correlated with higher depressive scores. Thombs BD et al. [17] using the Canadian Scleroderma Research Group registry showed that higher PGA scores, more tender joints, more GI symptoms, and more difficulty in breathing were disease-specific correlates of depression as were lower education level and being unmarried. Nietert PJ et al. [14] in a study conducted in the USA also showed that lower educational level correlated with depression symptoms as were worse GI functional status and worse disability. In a study by Golemati CV et al. [37], Greek SSc patients were compared to rheumatoid arthritis patients and healthy controls. When compared to the control group, SSc patients expressed more symptoms of depression and anxiety. They coped less often with positive reappraisal, problem-solving, seeking support and assertiveness, and they more often sought divine help. Higher disease activity, higher skin score, pulmonary hypertension, and lower FVC scores correlated with more depression symptoms. A lower education level and negative life events also correlated with depressive symptoms.

Levis B et al. [38] showed that depression on the CES-D correlated more with tender joints, worse GI symptoms, and more lung involvement. With regards to non-SSc predictors, they demonstrated that lower educational level and being unmarried also correlated to depression. Tedeschini E et al. in Italy [31], using the BDI showed that SSc-related factors such as disability, pain, disease activity, and articular involvement and non-SSc-related factors such as unemployment correlated with depression.

Matsura E et al. [18] looking at Japanese patients with SSc and using the BDI showed that a low sense of cohesiveness and increased helplessness are closely associated with depressive symptoms. Longer disease duration and pain were SSc-related predictors in a study by Ostojic P et al. [5] in Serbian patients using the BDI, while non-SSc-related predictors of depression were female sex, older age, unemployment, or retired status. In contrast, a study by Roca RP et al. [7] from 1996 showed that younger age correlated with increased depression as were the presence of digital ulcers, and worse self-related functional improvement based on the HAQ score.

A study from Iran using the BDI [41] revealed that the factors that related to depression in their population were a higher mRss, patients with dcSSc type of disease, and more pulmonary and GI manifestations. Lastly, a study by Wafki F et al. [44] in Morocco using the PHQ-9 showed that prolonged disease duration, severe joint pain, higher disease severity, and elevated inflammatory markers correlated with depressive symptoms.

## Discussion

Patients with chronic illnesses such as autoimmune rheumatic diseases suffer from mood disorders such as depression. The relationship between depression and chronic disease is bidirectional leading to worse long-term outcomes. Past research has shown that there is a higher prevalence of depression in SSc patients compared to other rheumatic diseases as mentioned previously [10, 11]. Depression is best diagnosed by a comprehensive clinical examination by a mental health professional and self-assessment questionnaires can be used as screening tools, especially for large populations of patients. However, these tools, when applied show variable prevalence [9]. In this systematic review, we limited our data collection to studies that have used three different self-assessment questionnaires to screen for depression (CES-D, BDI, and PHQ9) which have been validated in SSc patients [7, 29, 30]. Among all studies analyzed for this review, we found that 35.6% of SSc patients screened positive for depression. Interestingly when we sub-grouped the data based on the different questionnaires used, we noted that the CES-D and PHQ9 questionnaires had similar population prevalence of depression at 31.9% and 30.8% respectively while the BDI questionnaire in comparison showed a much higher prevalence at 55.1%. While this has important implications when used as a screening tool and both false positive and negative findings would need to be addressed, it could be that a higher BDI cutoff score may need to be used to better correlate with the cut-off of the other two instruments. The reasons for the wide variation in the prevalence of depression among various studies could be that the cut-off scores of each individual depression tool carries a different weight, differences in the design of each study as they were designed to answer different questions, country specific differences, and differences in patient demographics. Additionally, the presumed predictors of depression in each study were different and about 8 studies did not consider predictors of depression at all. We reported all the predictors of depression that reached significance in each individual study (Table 1) and categorized them together and presented them in a different table to better characterize them (Table 2). With all the disparities in study design, certain predictors of depression were found significant in a multitude of studies.

In five different studies, the severity of SSc-related gastrointestinal manifestations correlated with depressive symptoms. Gastrointestinal symptoms are among the leading clinical manifestations seen in SSc, with more than 90% of patients experiencing some sort of GI manifestations, often related to poor motility. While symptom-specific therapies are used for different GI manifestations, there is a lack of a unified therapy that addresses the motility issue specifically.

Pain, disease severity, and joint involvement in SSc patients carries with it significant disability, as many of the activities of daily living are affected by these symptoms. Therefore, it is not surprising that in multiple studies these symptoms correlated with higher depressive scores.

Regarding other SSc-related variables which met significance as predictors of depression in multiple studies, dyspnea was seen in three studies as was a higher mRss.

Non-SSc predictors of depression were looked out for in multiple studies. The one that stood out was lower educational level. In four studies included in this review, low educational levels correlated with higher depressive scores. The Norwegian study published in 2008 [50], including a large cohort of patients, found a significant association between low education levels and anxiety and depression. Moreover, unemployment and unmarried status were held as significant non-SSc predictors in two studies each, as was increased sensation of helplessness and less satisfaction with social support (Table 2).

Certain limitations are inherent with systematic reviews and were also a constraint in our analysis. Each study is designed to answer a different question and the way the SSc population is selected, depends on those questions. Thus, there was a significant selection bias, for example, one study was looking at marital satisfaction among married and unmarried women with SSc [38]. This type of bias may have impacted the selection process among all the studies included and the reported outcomes, thereby affecting the overall reliability of the findings.

SSc type seen across different countries varies widely. In the study from Morocco [44] included in the review 97% of patients had dcSSc, a more severe form of the disease, while in an Italian cohort [40] also used in our review only 24.3% of patients were dcSSc patients.

A major constraint on the results of this study is also how depression is regarded among different cultures and religions and differences in societal norms around the globe which are not captured with these studies, and we incorrectly assume that all these patients around the world are similar in how they see and deal with depression.

While existing research provides valuable insights into the potential drivers of depression in SSc, significant gaps remain regarding how these drivers should be addressed and the extent to which such interventions may be beneficial. More longitudinal studies are needed to compare patients' depressive states before and after improvements in these potential contributing factors. Additionally, pain has been identified as a significant predictor of depression in SSc. One study examined the effects of antidepressant therapy combined with a pain self-management intervention for patients with musculoskeletal pain and comorbid depression. The findings documented substantial reductions in depression severity, along with improved response and remission rates. [51] Although the study did not focus on SSc patients specifically, it provides valuable insight into the potential benefits of pain management, health education, and antidepressant therapy in addressing depression within this population. While our study has shown that GI involvement, pain, disease activity and pulmonary and joint symptoms correlate with depression, it is not clear if these parts of the disease are causative of depression or depression worsens these manifestations. In regard to pain management, one study looked at using opioids to treat pain from skin ulcers in SSc patients and they have shown based on a visual analog scale that pain improved and at the same time they also showed an improvement in sleep based on the Pittsburgh sleep quality index (PSQI) [52]. In a large population of older adults with osteoarthritis and comorbid depression, benefit of improved depression extended beyond this and included decreased pain, improved functional status and quality of life [53].

Assessment inventories used in papers provide insight into a patient’s depressive state but do not serve as a diagnostic tool for depression. As a result, there is limited information on the use of antidepressants in these patients. However, the study by Savoie (2023) reported that 90% of participants with probable depression had been clinically diagnosed with and/or treated for a mental health condition, and 85% of these participants received first-line pharmacotherapy for depression [35]. Antidepressants have been looked at in SSc but primarily in the treatment of Raynaud’s, showing a reduction in attack frequency and severity. Though studies directly targeting depression in this population are lacking, the fact that it may be able to target multiple symptoms in the same patients may warrant further research [54].

Based on the above work, we would recommend the following to manage depression when seeing SSc patients in the clinic: addressing modifiable indicators of depression, such as pain management and health education; integrating routine follow-ups with standardized assessment inventories for depression; ensuring close monitoring of patients with scores indicating possible depression; and facilitating early referral to a psychiatrist for those with scores suggesting probable depression.

## Conclusion

In conclusion, despite the constraints inherent with a systematic review, this study underscores and describes the prevalence of depression among a large cohort of SSc patients around the world, showcasing that there can be significant variability in estimates of depression among different self-assessment questionnaires. It also underscores the correlation of depression estimates in SSc-patients between the CES-D and PHQ9 scores at the cutoffs used for this study. It is important to also mention that since different self-assessment questionnaires are used for screening, resulting in various rates of false positives and false negatives that these patients need to be ultimately referred for a full clinical assessment by a mental health professional. It also highlights the importance that when certain predictors of depression in SSc-patients exist, such as significant GI and arthritic manifestations, increased pain and disease severity and a lower educational level that this should strengthen the need and willingness of mental healthcare professionals to engage in the care of patients as up to a third of SSc patients may benefit from this.
